# Artificial intelligence and virtual reality-based communication skills training in nursing education: a systematic review with implications for palliative care

**DOI:** 10.1186/s12912-026-05192-9

**Published:** 2026-08-07

**Authors:** James Raj K., Sharmila J.

**Affiliations:** 1 Department of Medical & Surgical Nursing, College of Nursing Sciences, Dayananda Sagar University, Bangalore, Karnataka India; 2 Department of Mental Health Nursing, College of Nursing Sciences, Dayananda Sagar University, Bangalore, Karnataka India

**Keywords:** Artificial intelligence, Virtual reality, Nursing education, Communication skills, Simulation, Palliative care

## Abstract

**Background:**

The need for communication is common to all nurses during patient interaction in different medical scenarios, such as palliative care and end of life care. It is important to note that many nurses and nursing students perceive their level of training as inadequate to deal with difficult conversations. The two tools – virtual reality and artificial intelligence – provide practical help in achieving this.

**Aim:**

To synthesize empirical evidence on artificial intelligence (AI)- and virtual reality (VR)-based communication skills training among nurses and nursing students and examine its implications for palliative care education.

**Design:**

Systematic review conducted in accordance with the Preferred Reporting Items for Systematic Reviews and Meta-Analyses (PRISMA) 2020 statement.

**Methods:**

Original studies written in English and published from database inception until March 2026 were identified. Studies that used AI- and/or VR-based communication training for nurses or nursing students using randomized controlled, quasi-experimental, mixed-methods, cross-sectional, or qualitative study designs were eligible for inclusion in this review. Thirteen studies were included in this review. Quality of the methodology was appraised using the Mixed Methods Appraisal Tool (MMAT).

**Results:**

Communication training through VR led to increased confidence in end-of-life communication, improved postmortem care knowledge, increased motivation and high acceptability. A 360 VR communication workshop was found to be feasible and scalable. Multiuser VR simulation reached the same communication outcome as live simulation, and VR-based communication training was more effective than video-based training for basic nurse-patient communication.

**Conclusions:**

AI- and VR-based communication training represents an innovative tool for nursing education, specifically in the field of palliative and end-of-life care. However, further randomized controlled trials with objective performance measurements and long-term follow-ups are needed due to the heavy reliance on subjective self-reports in the existing literature.

## Introduction

Communication is a fundamental and inextricable part of the nursing role, particularly as nurses serve as vital partners in the interdisciplinary team. Effective communication is one of the cornerstones of palliative care; it influences understanding of illness trajectories, trade-offs between options, and preparing for death [1–3]. In particular, nurses often act as mediators in palliative care and can serve as interpreters and/or advocates, communicating medical jargon, discovering values and preferences, and helping families through their crisis. However, many nurses feel unprepared to communicate about serious illnesses, particularly for providing bad news, addressing code-status decisions, and cultural/spiritual issues [3, 4].

Conventional training programs consist of theoretical learning, video-based teaching, simulation with role-playing exercises, and practice with SPs based on specific protocols like SPIKES and NURSE [1, 2]. This approach is effective yet requires many resources, faculty participation, and facilities; therefore, it cannot be easily used by practicing nurses in under-resourced countries [5, 6]. Digital-based approaches have been proposed as an alternative to conventional training; there is evidence from systematic reviews that e-learning and virtual simulations are effective in improving knowledge of palliative care and self-efficacy among nursing students; however, most of these solutions remain 2D and non-immersive [4, 7]. These conventional and 2D screen-based methods often fall short because they fail to realistically replicate the complex non-verbal cues, environmental context, and psychological pressure inherent in high-stakes palliative care conversations.

With recent developments in technology, VR and AI-based patient simulations have become more readily available for nurse education to address these exact training gaps [6, 8]. VR has the potential to generate authentic clinical environments and emotional contexts such as discussions regarding end-of-life care or code-status [9–11]. AI-powered simulations in the form of virtual counseling applications and conversational agents can simulate patient reactions, provide personalized feedback, and enable self-practice [12, 13]. With respect to palliative care, VR studies have also been conducted to investigate both pain management for patients and training of clinician-patient interactions [14, 15].

Although AI- and VR-based communication training has gained increasing attention in nursing education, the available evidence remains scattered across general nursing, interprofessional, serious illness, and palliative care settings. To date, no comprehensive systematic review has synthesized this evidence while examining its implications for palliative care nursing education.

### Research questions

1. What communication training interventions using AI or VR technology have been empirically tested in nurses or nursing students to improve communication skills, focusing on palliative care and other seriously ill patient settings?
2. How were the above interventions designed and implemented (e.g., type of scenario being addressed, level of immersion, whether using SPs or debriefing), and which types of study designs have been employed?
3. Which kinds of outcomes have been measured regarding communication skills or implementation feasibility?
4. What is the overall quality and certainty of evidence provided in terms of methodological rigor?
5. What implications or future directions does this research entail in terms of implementing these types of interventions within palliative-care nursing curricula and continuing education programs?

### Objective

The objective of this systematic review was to synthesize empirical evidence regarding artificial intelligence- and virtual reality-based communication skills training among nurses and nursing students and to examine its potential implications for palliative care education.

## Methodology

### Protocols and reporting standards

This systematic review was conducted and reported in accordance with the Preferred Reporting Items for Systematic Reviews and Meta-Analyses (PRISMA 2020) statement, which provides evidence-based guidance for reporting systematic reviews of healthcare interventions [16]. The review protocol was developed before literature searching and study selection commenced. The protocol was not prospectively registered; all review methods were predefined and consistently followed throughout the review process, in accordance with established recommendations for systematic reviews in nursing education and digital health research [8, 13, 16].

### Study design

This review employed a systematic review design to identify, critically appraise, and synthesize empirical evidence regarding artificial intelligence- and virtual reality-based communication skills training among nurses and nursing students. The review followed a predefined methodological framework guided by PRISMA 2020 recommendations and incorporated structured eligibility criteria, comprehensive database searching, quality appraisal, and narrative synthesis because of methodological heterogeneity across the included studies [16].

### Eligibility criteria

The eligibility criteria were specified based on the PICOS approach, following earlier reviews on digital palliative care education and VR training for communication skills among health professions [4, 7].

#### Population

The participants comprised registered nurses, new graduates, and pre-registration nursing students. In addition, mixed groups of learners, such as nurses and medical students, were regarded as eligible where there was at least some representation of nurses and where the scenario involved communication with seriously ill or dying patients.

#### Intervention

Interventions that used VR in any form including immersive VR, 3D serious games, and 360° video simulations that entailed nurse-patient or interprofessional communication in palliative care/end-of-life care or serious illness/foundational therapeutic communication were included. Interventions that used AI in virtual counseling tools, virtual patients, and AI-enhanced VR environments for training communication skills in nursing were also considered.

#### Comparators

Comparator was accepted, whether it was a traditional teaching method, non-virtual reality (VR) digital media, video teaching methods, live simulations, or pre-test/post-test within-subject designs without a specific control group. The comparator conditions were defined a priori to include all the educational methods that have been employed thus far in communication skills training.

#### Outcomes

The communication outcome measures included self-efficacy (or confidence) for communication, communication knowledge, communication skill performance (such as checklist scores or standardized patient ratings), teamwork or inter-professional communication, counseling skills, and other similar measures. Measures of implementation outcomes such as feasibility, usability, acceptability, satisfaction, intention to use, and barriers/facilitators were also included.

#### Study types

RCTs, quasi‑experimental studies, mixed‑methods studies, cross‑sectional surveys, and qualitative descriptive designs.

#### Other criteria

Only those articles where humans were used as subjects of research and written in the English language were considered in the review. Empirical studies that presented findings were chosen, whereas conference abstracts, protocol papers, and theoretical papers that did not present any findings were not included in the review. Reviews, systematic reviews, and meta-analysis papers were not taken as primary sources of data but used for discussing findings.

### Search strategy and databases

A comprehensive literature search was undertaken in MEDLINE/PubMed, Embase, CINAHL, PsycINFO, Scopus, and Web of Science from database inception to March 2026. Additional searches included hand-searching reference lists and citation tracking of relevant reviews on AI- and VR-based communication training [6, 13]. The exact same key concepts were searched for in each database; however, the syntax was modified according to each database’s index terms, subject headings, field codes, etc. In addition, controlled vocabulary, along with keywords, was employed to get the best possible results.

The search strings consisted of controlled vocabulary and keyword terms for palliative/end-of-life care, nursing, communication, and VR/AI. The search strategy used is:

(“palliative care” OR “hospice” OR “end‑of‑life” OR “serious illness”) AND.

(“nurse” OR “nurses” OR “nursing student*”) AND.

(“communication” OR “breaking bad news” OR “goals of care” OR “advance care planning”) AND.

(“virtual reality” OR “VR” OR “virtual simulation” OR “360‑degree video” OR “virtual patient” OR “conversational agent” OR “chatbot” OR “artificial intelligence”).

Other search terms, including “therapeutic communication,” “interprofessional communication,” and “team training,” were added in order to encompass communication training more broadly relevant to palliative-care practice [11, 17]. All the searches performed were captured in a systematic search log where the database/platform, date of search, search term, filters applied, and the number of records captured were noted. The records were then exported to reference manager software for organizing and eliminating any duplicates. For reporting under the PRISMA guidelines, records obtained from each of the databases and those obtained via the supplementary historical searches were noted individually before any duplication. The historical search was done by going through the references of included articles and reviews as well as citation tracking of articles captured during the initial search in the databases.

### Screening process

The selection of titles and abstracts was done using the eligibility criteria, while full-text papers of those that appeared to meet the eligibility requirements were selected using the PRISMA 2020 guidelines [16]. The title/abstract screening and full text selection were done independently by two reviewers following the specified inclusion criteria. Where there were any differences of opinion concerning inclusion of studies, these were sorted out through discussion, while unresolved issues were taken up with a third reviewer. From this selection, 13 empirical studies were identified to be included in this review.

### Data extraction and management

For each selected article, the following information was collected: author(s), year, location, population, sample size, setting, clinical topic (end-of-life care, serious illness, general), VR vs. virtual patient/technology, scenario content, duration, structure including debriefing, comparison group (if any), outcome measures and tools used, findings, and limitations [7, 13].

The data collected from the literature review were compiled into tables for consistency and accuracy compared to the original articles [18]. A standardized process for data extraction was employed to facilitate consistency in data reduction and to guarantee that inclusion of the selected studies in the synthesis process matched the predefined PICOS criteria. Determinations about data classification and reduction were made on the basis of comparisons among the interventions, participants, and outcomes from different studies.

### Risk of bias assessment

Assessment of risk of bias was performed using the MMAT 2018 tool, which provides design-specific criteria for qualitative studies, randomized studies, non-randomized studies, descriptive studies, and mixed-methods research [18]. Two independent reviewers who were involved in screening and synthesis phases (the reviewer and this synthesis process) evaluated the MMAT criteria for each study, including selection bias, measurement bias, confounding, outcomes reporting, and mixed methods consistency [18]. Results are summarized narratively and in Table 4.

### Data synthesis and analysis

Due to heterogeneity of the interventions and outcomes and low numbers of studies per outcome domain, pooling was not attempted quantitatively. Narrative synthesis was used for this meta-review, with studies grouped into three categories:

1. Approaches focused on explicitly palliative or end-of-life care;
2. Severely ill patients or interprofessional communication initiatives; and.
3. General therapy and counseling in nursing training.

The effects observed in each domain were subsequently mapped out and qualitatively evaluated for evidence certainty through the GRADE approach [19, 20]. The results are summarized in Tables 2 and 3.

### Reporting bias assessment

A formal assessment of reporting bias (e.g., publication bias using funnel plots or statistical tests) was not performed because only 13 heterogeneous studies were included and no meta-analysis was conducted. Consistent with PRISMA 2020 recommendations, assessment of reporting bias was considered inappropriate due to the limited number of studies and substantial methodological heterogeneity.

## Results

### Study selection

**Fig. 1:**
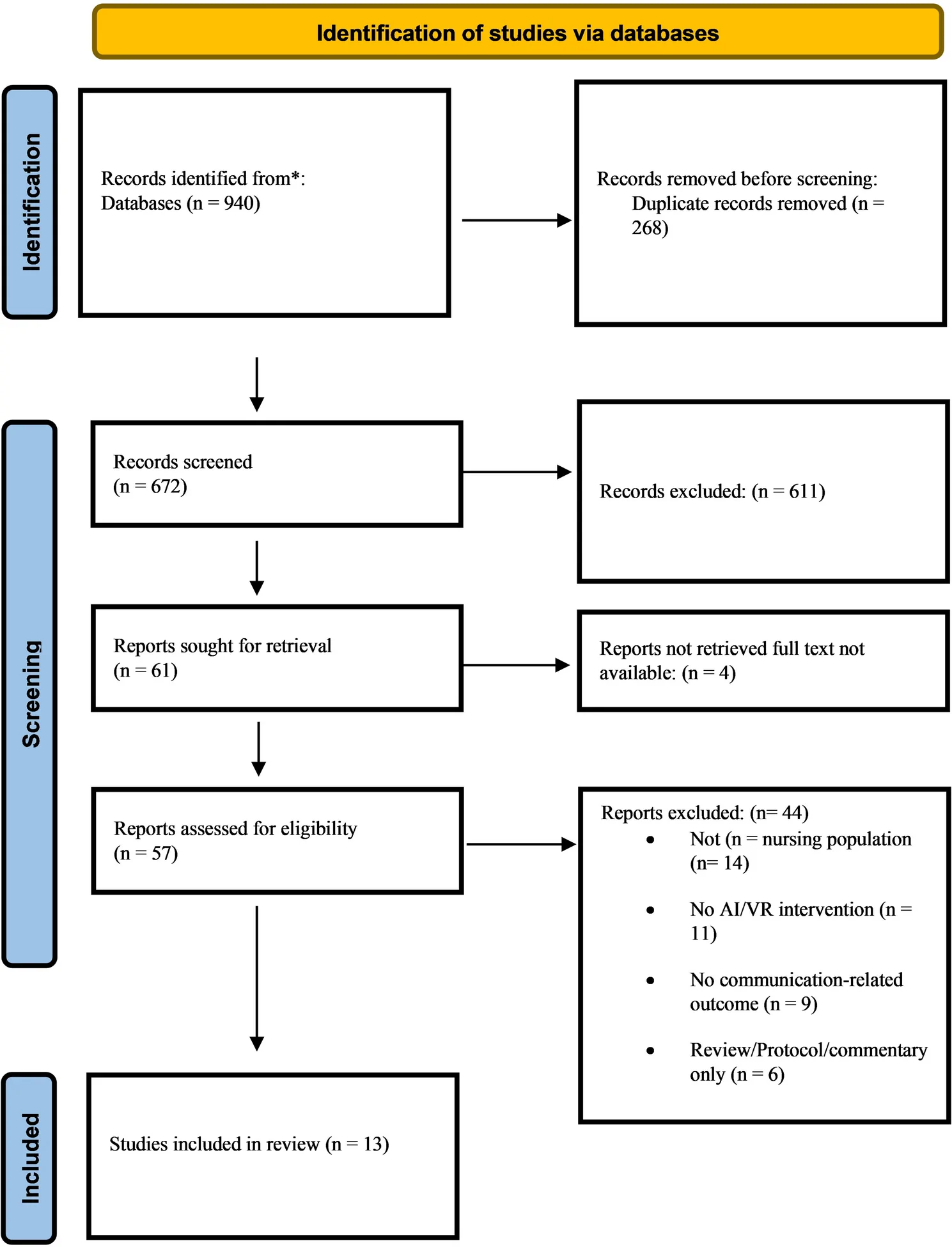
PRISMA 2020 flow diagram

A total of 13 studies met the predefined inclusion criteria and were included in this systematic review. Full-text articles excluded during eligibility assessment were primarily those involving ineligible study populations, interventions unrelated to AI- or VR-based communication training, non-empirical study designs (e.g., reviews or protocols), conference abstracts, or insufficient outcome data. The complete study selection process is presented in the PRISMA 2020 flow diagram (Fig. 1).

### Study characteristics

Criteria for inclusion of studies assessing virtual reality, artificial intelligence, and virtual patient-based interventions for developing communication skills in nursing and interprofessional education programs. Table presents information regarding settings, participants, focus on clinical communication, intervention features, and study designs of the selected literature related to palliative care, end-of-life communication, counseling, and teamwork among various disciplines. Selected studies reflect different educational settings that utilize technology-supported simulations to improve communication skills in nursing education and practice (see Table 1 below).

**Table 1:** Characteristics of included AI-, VR-, and virtual patient-based communication training studies involving nurses or nursing students (*n* = 13), presented alphabetically by first author

Study	Country	Population & sample	Clinical focus	Technology & intervention	Design
Chen et al. (2026) [21]	Taiwan	Newly employed nurses and nursing students at eight schools (*n* ≈ 1,789)	Post‑mortem care as key EOL nursing task	VR post‑mortem care module (body preparation, clothing, grooming, positioning) delivered via head‑mounted display	Quasi‑experimental one‑group pre–post
Chou et al. (2024) [25]	Taiwan	Nursing students (*n* = 84; 42 VR, 42 video)	Fundamentals of nurse–patient communication	VR communication simulation (HTC Vive Focus 3) vs. communication video in fundamentals practicum; four measurement points	Pragmatic randomized controlled trial
Hall et al. (2024) [9]	Australia	3rd‑year nursing students (*n* ≈ 79)	End‑of‑life care; ACP and EOL communication	Low‑cost VR (VirtualU End‑of‑Life Care) experienced via Google Cardboard, followed by SP encounters and structured debriefing	Single‑arm repeated‑measures pre–post
Hara et al. (2021) [28]	Brazil	Nursing professors and students	Communication learning and competence	3D immersive VR serious game “Comunica‑Enf” using Oculus Rift; usability, suitability for communication training	Methodological quantitative study
Hwang et al. (2022) [27]	Taiwan	Senior nursing students (*n* = 40; VP vs. control)	Clinical judgment and communication in nursing care	Virtual‑patient‑based social learning platform vs. conventional instruction; performance, self‑efficacy, communication assessed	Quasi‑experimental
Liaw et al. (2020) [17]	Singapore	Medical and nursing students (*n* = 120)	Nurse–physician communication about deteriorating patients	Multi‑user VR acute‑care scenario vs. conventional team simulation; nurse–physician teams; communication and teamwork assessed	Randomized controlled trial
Liaw et al. (2023) [24]	Singapore	Final‑year nursing students (*n* = 32)	Interprofessional communication and telehealth	AI‑enabled VR simulation with AI doctor agent and virtual patients in a 3D ward; pre–post knowledge and self‑efficacy	Mixed‑methods single‑group
Lin et al. (2026) [22]	East Asia (high‑context culture)	Practicing nurses (*n* ≈ 50)	End‑of‑life care education in high‑context cultures	Immersive VR (IVR) EOL care education module; pre/post knowledge tests and qualitative feedback	Mixed‑methods pre–post
Ouchi et al. (2023) [10]	USA	Interprofessional clinicians including nurses	Palliative‑care communication; code‑status and goals‑of‑care discussions	360° VR adaptation of an evidence‑based palliative‑care communication workshop, reducing a 4‑hour actor‑based course to a 1‑hour VR session	Program description and feasibility evaluation
Saab et al. (2026) [11]	Five European countries	Nursing and medical students (*n* ≈ 268)	“Challenging communication” scenarios in serious‑illness care	Immersive VR communication scenario (VR‑TALKS) delivered via headsets; feasibility, usability, satisfaction questionnaires	Cross‑sectional multi‑country study
Shorey et al. (2019) [12]	Singapore	Undergraduate nursing students (*n* ≈ 160)	Counseling and therapeutic communication	AI‑based virtual counseling application simulating patient–nurse counseling sessions; multi‑semester use	Development and evaluation study
Shorey et al. (2020) [26]	Singapore	Nursing students and clinical facilitators	Experiences of VR communication training	VR communication simulation; focus groups and interviews explored perceived benefits and limitations	Descriptive qualitative study
Skvortsova et al. (2025) [23]	Multiple European countries	Health‑professions educators (*n* ≈ 75)	Use of VR for serious‑illness communication education	Online survey using ADOPT‑VR scales assessing attitudes, perceived usefulness, norms, and intention to use VR	Cross‑sectional survey

Table 1 indicates that most interventions came from high-income countries, with significant contributions coming from East Asia, Europe, and Singapore-based studies, with most using undergraduate nursing students as participants, but there were two exceptions involving practicing nurses [21, 22].

### Distribution of studies by design

**Fig. 2:**
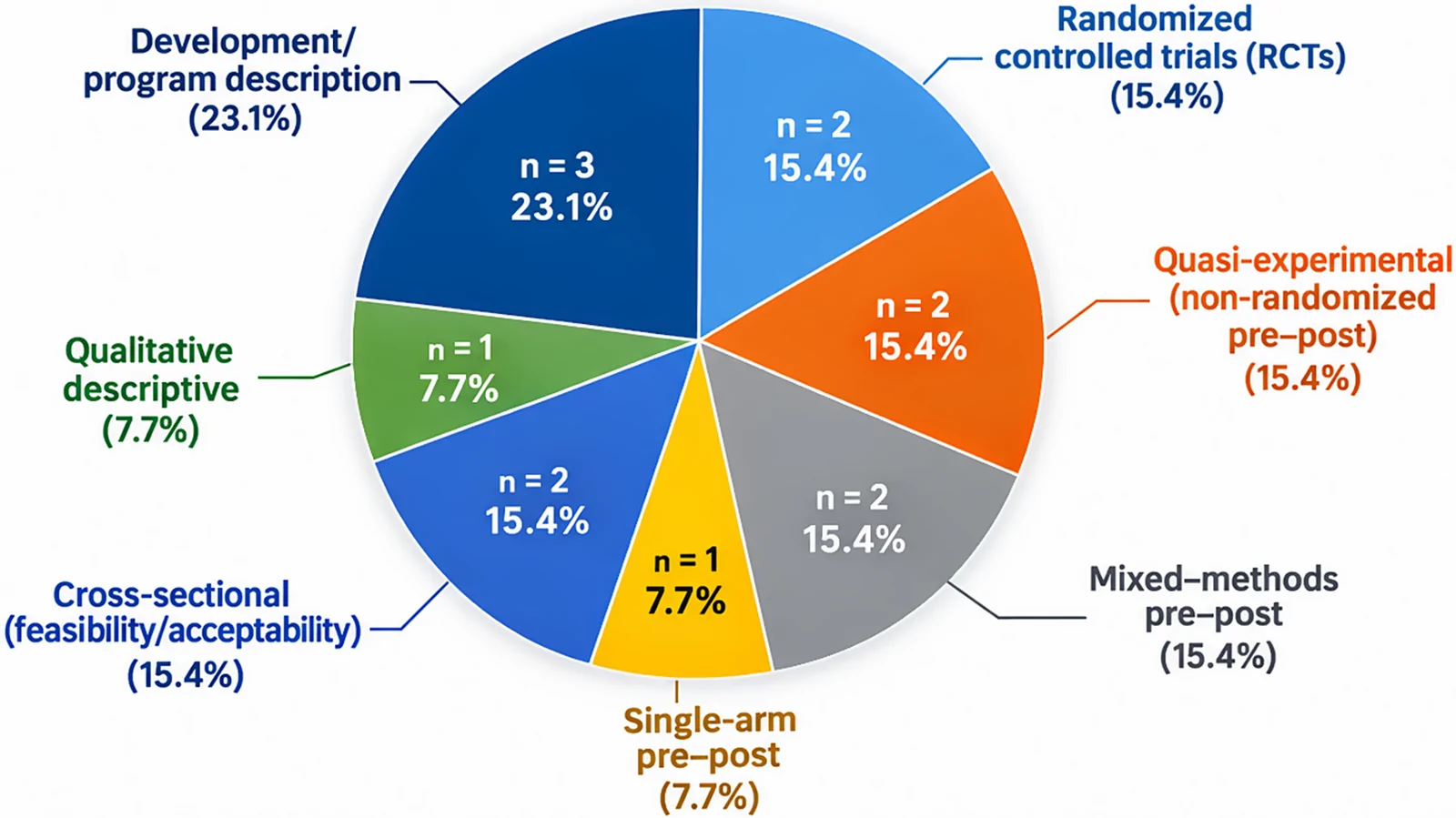
Distribution of included studies by design type

The types of designs used by the included studies varied widely. Two studies adopted randomized control trial designs, whereas the other two studies used non-randomized pre–post designs. A total of two mixed methods pre–post design studies were adopted, whereas a single-arm pre-post design was used in one study. Cross-sectional studies were adopted in two studies, which mainly focused on the feasibility and acceptability aspects. A qualitative descriptive study was adopted in one study, and three studies were classified under development or program description studies. The distribution of studies is represented in Fig. 2.

### End‑of‑life and palliative‑care–focused interventions

According to Hall et al. (2024), a scaffolded intervention comprising a cost-effective VR module dealing with the end-of-life issues together with simulation exercises resulted in significant improvements in the self-assessment of confidence related to 13 end-of-life communication skills, such as communication about death and dying, cultural and spiritual aspects, and advance care planning for third-year nursing students [9]. Specific values of effect size are not reproduced, but one can say that all 13 items were significantly improved (*p* < 0.05) with moderate to high effect sizes, and the qualitative data demonstrated that the use of VR made the situation easier, setting the stage for more difficult simulation sessions.

Chen et al. (2026) observed that VR-based post-mortem care training led to significant gains in knowledge of post-mortem care among both newly hired nurses and nursing students; however, the improvement was especially notable among those without prior hospice experience. Apart from gaining knowledge, trainees felt ready and less nervous about performing post-mortem care, which constitutes an important part of end-of-life nursing practice [21].

Lin et al. (2026) conducted a study on the use of IVR in educating practicing nurses regarding end-of-life care within a high-context culture wherein discussion of mortality is not always openly permitted. According to their findings, there was a considerable enhancement in their knowledge regarding end-of-life care, and they also scored highly in terms of satisfaction, usefulness, and motivation. In this study, they valued their exposure to the situation both as patients and their families [22].

The work of Ouchi (2023) involved a 360° VR adaptation of an evidence-based palliative-care communication training program that enabled medical professionals such as nurses to experience goal-of-care discussions within the span of only an hour rather than four hours in a typical live setting [10]. Although no formal results had been obtained, the pilot testing indicated promising findings in terms of feasibility and scalability.

### Serious‑illness and interprofessional communication

In a cross-sectional study of multiple countries, Saab et al. (2026) found high levels of usability, feasibility, and satisfaction with a VR communication scenario for patients facing serious illnesses among nursing and medicine students in five European countries [11]. The participants valued the simulation’s realism as well as the ability to practice challenging communication situations, but there was no pre/post assessment of skills.

Skvortsova et al. (2025) conducted a survey of health professions instructors regarding their perceptions of using VR to teach serious illness communication and discovered positive attitudes towards it as well as perceived usefulness of the tool, but relatively low adoption intentions due to concerns regarding costs, technology support, and curriculum compatibility [23].

According to Liaw et al. (2020), in a randomized control trial, multi-user VR was compared to live simulation regarding improving the communication between nurses and physicians in regard to patient deterioration. There were substantial improvements in team communication performance and teamwork attitudes in both groups, with no significant differences observed in the VR and live simulation, meaning that VR can serve as a good alternative option for training some interprofessional communications [17].

In another study by Liaw et al. (2023), there was a creation of AI-enabled VR simulation with interaction of final-year nursing students with AI doctor agents and virtual patients in 3D ward setting. Improvements in knowledge and self-efficacy of communication along with positive views on usability and realism were achieved; however, due to the nature of research (pre-post single group design), causation cannot be assumed [24].

### General therapeutic‑communication and counseling training

The study by Chou et al. (2024) was a pragmatic randomized controlled trial that involved comparing VR communication simulation and a traditional communication video in the fundamentals of nursing practicum [25]. Students using VR demonstrated better communication competency and confidence, learning satisfaction, and low clinical stress during several follow-ups as compared to the control group, yielding moderate evidence for the potential of VR to improve essential communication competencies of nurses as compared to video-based learning.

Shorey et al. (2019) introduced an AI-based virtual counseling program and evaluated its impact on nursing students’ practice. The learners found counseling skills training beneficial, gained self-confidence, and positively valued the opportunity to practice at their own speed. Educators recognized the application as an inexpensive way to support clinical practice [12].

In another study conducted to evaluate the effectiveness of VR in enhancing communication skills, Shorey et al. (2020) interviewed the learners and facilitators of VR communication training and discovered that there were issues in terms of the realism of the avatars used and in the use of language; nevertheless, the participants reported greater engagement and confidence [26].

The effectiveness of virtual patients was tested by Hwang et al. (2022), who found that the group with virtual patients performed better than the control group with traditional learners, as far as learning performance, self-efficacy, and communication skill improvement were concerned [27].

Another study involving 3D serious games for training patients’ communication skills is that of Hara et al. (2021). They developed and evaluated the 3D serious game called “Comunica-Enf” that proved highly effective in terms of usability and applicability [28].

### Outcome markers, recommendations, and GRADE

Summary of research evidence on the use of VR, AI, and virtual patient-based interventions for improving communication training in nursing and interprofessional education. This table includes outcome domains, important findings, suggestions for implementation in practice, and the GRADE level of certainty based on the research evidence pertaining to end-of-life communication, counseling, interprofessional collaboration, clinical decision-making, and serious illness communication. In general, the research evidence indicates that simulated learning via virtual reality and AI has the potential to improve aspects of confidence, engagement, and self-efficacy in communication training (Table 2).

**Table 2:** Outcome markers, recommendations, and GRADE certainty for AIVR communication-skills training in nursing (*n* = 13)

Outcome domain	Evidence (studies)	Main findings	Practice recommendation	GRADE certainty*
End‑of‑life communication confidence in students	Hall et al. 2024 [9]	VR + SP + debriefing produced significant pre–post gains in self‑reported confidence across 13 end‑of‑life communication items; students valued VR as preparatory exposure.	Incorporate scaffolded VR + SP programs into undergraduate palliative‑care curricula as an adjunct to standard teaching.	Low
Post‑mortem care knowledge and preparedness	Chen et al. 2026 [21]	Significant improvement in post‑mortem care knowledge, especially among participants without prior hospice training; perceived preparedness improved.	Use VR post‑mortem care modules to standardize and strengthen EOL nursing education for novice nurses and students.	Low–moderate
End‑of‑life care knowledge and motivation (practicing nurses)	Lin et al. 2026 [22]	IVR EOL module improved knowledge and motivation; nurses rated the experience highly and viewed it as a valuable supplement.	Offer IVR EOL modules as feasible, acceptable additions to in‑service palliative‑care training, particularly in high‑context cultures.	Low
Palliative‑care communication dissemination	Ouchi et al. 2023 [10]	360° VR adaptation reduced resource demands of a palliative‑care communication workshop and showed good feasibility, but learning outcomes not yet reported.	Pilot VR‑based dissemination of palliative‑care communication curricula and evaluate learning outcomes before large‑scale implementation.	Very low
Foundational communication ability and stress	Chou et al. 2024 [25]	VR group outperformed video control on communication ability, confidence, learning satisfaction, and had lower clinical stress.	Use VR communication simulations to enhance foundational nurse–patient communication as part of core curricula.	Moderate
Team communication and teamwork attitudes	Liaw et al. 2020 [17]	VR and live‑simulation groups showed similar improvements in team communication and teamwork attitudes.	Consider multi‑user VR as a viable alternative or complement to live simulation for interprofessional communication training.	Moderate
Interprofessional communication knowledge and self‑efficacy with AI‑VR	Liaw et al. 2023 [24]	AI‑enabled VR simulation improved communication knowledge and self‑efficacy; high perceived usefulness and realism.	Use AI‑enabled VR as an adjunct for interprofessional and telehealth‑related communication training, with careful evaluation of content accuracy.	Low
Counseling/therapeutic communication skills (AI virtual counselor)	Shorey et al. 2019 [12]	AI virtual counseling app enhanced communication skills and confidence; students appreciated repeated practice.	Integrate AI virtual counseling tools as supplementary practice resources, with faculty oversight and iterative refinement.	Low
Learner experience and engagement with VR	Shorey et al. 2020 [26]	Students reported greater engagement and confidence but noted limitations in avatar realism and language.	Combine VR training with structured debriefing and real‑world clinical exposure; update VR content regularly.	Very low
Clinical judgment and communication with virtual patient	Hwang et al. 2022 [27]	VP‑based social learning group showed better performance and self‑efficacy than control; improved communication and clinical judgment.	Use virtual‑patient platforms where VR hardware is limited, focusing on communication and decision‑making.	Low–moderate
Communication competence and usability of serious game	Hara et al. 2021 [28]	Comunica‑Enf serious game rated usable and suitable; contributes to communication learning but outcome measures limited.	Employ immersive serious games as engaging supplements to traditional communication‑skills teaching.	Very low
Usability and acceptability of VR serious‑illness communication	Saab et al. 2026 [11]	High usability and satisfaction among students; minimal adverse effects.	Integrate VR serious‑illness communication scenarios into curricula, particularly for early exposure to challenging conversations.	Low
Educator intention to adopt VR for serious‑illness communication	Skvortsova et al. 2025 [23]	Educators perceive VR as useful but face barriers (cost, infrastructure, training); intention to adopt is moderate.	Address resource and training barriers and integrate VR into existing communication curricula rather than implementing as stand‑alone.	Low–moderate

**Abbreviations**: AIVR = artificial intelligence- and virtual reality-based; AI = artificial intelligence; VR = virtual reality; VP = virtual patient; EOL = end-of-life; ACP = advance care planning; SP = standardized patient; GRADE = Grading of Recommendations Assessment, Development and Evaluation. GRADE is a framework used to rate the certainty of evidence across outcome domains by considering study design, risk of bias, inconsistency, indirectness, imprecision, and publication bias

### GRADE certainty profile

GRADE grading of the certainty of evidence from VR-, AI-, and virtual patient communication training programs in nursing and interprofessional education, as well as the basis for downgrading the quality of evidence (Table 3). Certainty of evidence was assessed qualitatively based on GRADE recommendations [19, 20].

**Table 3:** Summary GRADE certainty profile for selected outcome domains (*n* = 13)

Outcome domain	Studies	Consistency	Overall certainty	Main reasons for downgrading
EOL communication self‑efficacy (students)	Hall et al. 2024 [9]	Single study	Low	Non‑randomized design; self‑report; single institution.
EOL/post‑mortem care knowledge	Chen et al. 2026; Lin et al. 2026 [21, 22]	Generally consistent	Low–moderate	Non‑randomized designs; limited settings; short‑term follow‑up.
Interprofessional team communication performance	Liaw et al. 2020; Liaw et al. 2023 [17, 24]	Consistent direction	Moderate	RCT for VR vs. live sim; AI‑VR evaluated only in single‑group design.
General communication ability and stress	Chou et al. 2024 [25]	Single RCT	Moderate	Student sample; one context.
VR usability/acceptability for serious‑illness communication	Saab et al. 2026; Skvortsova et al. 2025 [11, 23]	Consistent	Low	Cross‑sectional designs; self‑selection; no objective skill measures.
Therapeutic/counseling skills with AI/VP	Shorey 2019; Hwang et al. 2022; Hara et al. 2021 [12, 27, 28]	Generally positive	Low	Non‑randomized designs; small samples; indirectness to palliative care.

Abbreviations: GRADE = Grading of Recommendations Assessment, Development and Evaluation; RCT = randomized controlled trial; VR = virtual reality; AI = artificial intelligence; VP = virtual patient; EOL = end-of-life; SP = standardized patient. GRADE is a framework used to rate the certainty of evidence across outcome domains by considering study design, risk of bias, inconsistency, indirectness, imprecision, and publication bias

### Risk of Bias and Quality

The risk of bias across the studies is presented in Table 4. To summarize, RCTs usually had low selection bias but still were prone to problems with blinding and single-site students sampling, whereas non-randomized and descriptive research was usually performed without controls and with the use of convenient sampling and self-reports [18].

**Table 4:** Risk-of-bias overview using MMAT 2018 (*n* = 13)

Study	Design	Selection bias	Measurement / performance bias	Attrition / reporting bias	Other concerns	Overall risk
Hall et al. 2024 [9]	Single‑arm pre–post	High (convenience sample, no control)	High (unblinded, self‑report)	Low–moderate	Single site; social desirability	High
Chen et al. 2026 [21]	Quasi‑experimental	High (no control group)	Moderate	Low	Potential confounding (prior training, institutional differences)	High
Lin et al. 2026 [22]	Mixed‑methods	Moderate	Moderate	Low	Cultural specificity; short‑term follow‑up	Moderate
Ouchi et al. 2023 [10]	Program description	High	High	High	No formal outcome assessment	Very high
Saab et al. 2026 [11]	Cross‑sectional	High (voluntary, multi‑site)	Moderate	Low	Lack of performance outcomes	High
Skvortsova et al. 2025 [23]	Cross‑sectional	High (snowball sampling)	Moderate	Low	Self‑report; no behavioural data	High
Chou et al. 2024 [25]	RCT	Low (randomization described)	Moderate (no blinding)	Low	Single‑country student sample	Moderate
Liaw et al. 2020 [17]	RCT	Low	Moderate	Low	Simulation performance; limited real‑world outcomes	Moderate
Liaw et al. 2023 [24]	Mixed‑methods	Moderate	Moderate	Low	Single‑group pre–post; small sample	High
Shorey et al. 2019 [12]	Development study	High (convenience sample)	High (self‑report)	Low	Limited generalizability; early‑stage tool	High
Shorey et al. 2020 [26]	Qualitative	Moderate	N/A	Low	Single institution; small sample	Moderate
Hwang et al. 2022 [27]	Quasi‑experimental	Moderate	Moderate	Low	Modest sample size; potential confounding	Moderate–high
Hara et al. 2021 [28]	Methodological	High	High	Low	Focus on usability; minimal performance data	High

Abbreviations: MMAT = Mixed Methods Appraisal Tool; RCT = randomized controlled trial; VR = virtual reality; AI = artificial intelligence; VP = virtual patient; EOL = end-of-life; SP = standardized patient. GRADE is a framework used to rate the certainty of evidence across outcome domains by considering study design, risk of bias, inconsistency, indirectness, imprecision, and publication bias

## Discussion

### Summary of key findings

This systematic review identified 13 empirical studies evaluating artificial intelligence- and virtual reality-based communication skills training among nurses and nursing students. Although only a limited number of studies specifically focused on palliative or end-of-life care, the overall evidence consistently demonstrated that immersive technologies improved communication confidence, learner engagement, self-efficacy, communication performance, and educational satisfaction across different nursing education settings. The strongest evidence was observed for virtual reality-based simulation, whereas evidence supporting AI-assisted communication training remains limited but encouraging [9, 12, 17, 21, 22, 24, 25]. Significantly, these results have addressed the objectives of the review in that they have illustrated not only the technologies which have been used in research, but also the consistent benefit which they have provided in terms of communication rehearsal and preparation rather than clinical outcomes.

Thus, VR-based end-of-life training increased nurses’ and nursing students’ confidence as well as nurses’ end-of-life knowledge and motivation [9, 21, 22]. Interprofessional communication through VR proved to be equally effective as live simulations [17], and VR communication simulation was more effective than video-based training in a fundamentals practicum [25]. VR applications enhanced communication knowledge, self-efficacy, and counseling skills among nursing students with the use of AI [12, 24, 27]; similarly, communication learning as well as clinical decision-making were possible with the help of VR serious games and virtual patient models. Both educators’ and students’ feedback on the use of VR in communication training indicated high acceptance and cost considerations [11, 23]. In totality, the research indicates that VR is the more developed and more validated technology of the two, with the use of AI being a more nascent idea in education, especially in the context of palliative care.

In general, though, the degree of certainty associated with the evidence varies between being very low and moderate, with the strongest level of evidence being found regarding the use of VR technology as a means for improving foundational and interprofessional communication, followed by less strong evidence regarding AI-based tools and applications for the palliative care setting. This grading pattern helps to interpret the results because it enables one to identify whether there is any intervention which has the initial feasibility support or whether there is another intervention which has relatively more effect on education.

### Interpretation and mechanisms

In combination with previous research findings, the current data suggest that simulation-based education techniques such as VR and virtual patients may enhance communication skills, increase self-efficacy, and promote learner engagement in healthcare education programs [6, 8, 13, 29]. As a tool capable of immersing learners into a highly engaging yet psychologically safe environment, VR seems especially useful for simulating the challenging context associated with serious illness and palliative care, in which learners need practice responding to distress, uncertainty, and value complexity [9–11]. This analysis is consistent with the review objective of making clear how these technologies can provide a link between theoretical education and emotionally taxing real-world communication.

Specifically, VR may facilitate learning through mechanisms such as increased presence and emotional engagement, scaffolding for exposure to challenging communication, and repeated exposure to consistent situations [30, 31]. In Hall’s study, students viewed the VR module as a “warm-up,” helping them get used to the concept of end-of-life care before facing higher fidelity SP interactions—a strategy also supported by existing literature on how learners should gradually develop competencies for emotionally challenging situations [1, 9]. Similar conclusions may apply to Lin’s IVR simulation, which provided opportunities for nurses in a high-context culture to “step into” end-of-life scenarios that may not occur in daily life [22]. This implies across all the studies that VR acts not as a substitute to human tutoring but more as a preparation phase for simulation and real clinical interaction.

Virtual counseling and virtual patient systems using AI have further benefits, including the ability to practice and receive feedback as and when needed [12, 13, 27]. Virtual tools can help to develop basic communication skills that form the foundation of palliative care consultations, even if particular situations do not necessarily fall within the ambit of palliative care [1, 2]. Qualitative research suggests that students are sensitive to any lack of realism in avatars, language, and emotion, which is an important point to consider in designing these systems [15, 26]. This limitation is especially relevant for palliative care, where authenticity, empathy, and cultural sensitivity are central to effective communication.

### Comparison with existing literature

Our results corroborate and expand on the existing reviews of VR in nurse education and training communication through virtual patients. Plotzky et al. (2021) systematically reviewed VR simulations in nurse education and found substantial interest in VR for communication and clinical decision-making; however, there were relatively few palliative care applications [6]. Shorey & Ng (2022) noted that VR simulations positively affect learning outcomes and learner satisfaction in nurse education, but pointed out the need to address issues regarding the methodology quality and duration of assessments [8]. Lane et al. (2020) conducted a systematic review of virtual patient simulators for medical communication and found that adequately designed simulators can successfully boost communication effectiveness; however, some simulators lack evaluation and emotional realism [13]. This review expands on the literature by examining the topic of interest specifically with regard to nurses and nursing students, and through a synthesis of evidence from palliative care, interprofessional care, and general communication literature all in one framework.

In terms of palliative care applications of VR, Mo et al. (2022) identified that existing research mainly concentrated on symptom management in patients, with little evidence regarding education for clinicians; the authors recommended additional rigorous studies concerning the efficacy of using VR in educating about palliative care communication [14]. Similarly, Schenker et al. (2024) considered conversational agents in palliative care settings and outlined the future research agenda that would encompass the examination of safety and ethical concerns, as well as potential applications to educate clinicians [15]. This review identified very limited empirical evidence regarding AI conversational agents specifically developed for communication training in palliative care nursing. Existing evidence mainly relates to broader nursing communication education, highlighting an important opportunity for future research in palliative care–specific educational applications [12, 15, 31, 32]. This gap is critical as it indicates that the field has promising interventions, but does not have adequate palliative care-specific evidence to facilitate implementation.

### Strengths of the present review

Some of the strong points of this review include that it incorporates both AI and VR technologies into its analysis on the spectrum from fundamental communications to specifically palliative care communications in order to provide an overview of the technological trend in the field of nursing communication education. Second, the fact that the review was tailored to specifically look at the nurse and nursing student populations is important since it is vital to consider the critical role nurses play when discussing palliative care communications. Third, frameworks such as PRISMA 2020, MMAT, and GRADE were utilized [16, 18–20]. Further, the review brings together educational impacts, implementation factors, and evidence certainty, enabling the results to speak to curriculum development and research directions.

### Limitations

However, there are several important considerations that should limit interpretation. First, only three papers focused on end-of-life care/palliative care and did not utilize a randomized design among practitioners in palliative-care nursing; the inferences about the usefulness of the research results for training in palliative care largely depend on the analysis of students’ experiences and/or the results obtained during training on communication skills [9, 21, 22]. Second, in most studies, the outcomes were subjective (self-reported experience, self-efficacy, confidence) and were assessed in the short term without measuring learners’ performances and analyzing their results after long-term practice [17, 25, 27]. This means that the review can support short-term educational value more confidently than durable practice change or patient-level benefit.

Third, significant diversity of the interventions used, learner characteristics, and outcome measures prevented the use of the meta-analysis and identification of key factors facilitating successful learning through VR/AR based technologies [5, 6, 13]. Fourth, in many papers, there was an evident high risk of bias in terms of methodology; in particular, in non-randomized and descriptive studies [18]. Fifth, many VR/AR-based interventions were developed and tested within single centers, making them less generalizable [29, 33]. Sixth, our systematic literature review was limited to English-language articles and papers available up to March 2026. Together, these limitations mean that the current evidence base should be interpreted as promising but preliminary, especially for palliative-care-specific applications.

Another limitation is that most included studies evaluated undergraduate nursing students rather than practicing palliative care nurses. Therefore, the findings should be interpreted as evidence supporting communication education in nursing broadly, with indirect implications for palliative care nursing practice. In addition, considerable heterogeneity in intervention characteristics, educational settings, and outcome measures limited direct comparison across studies and prevented quantitative meta-analysis [5, 6, 13]. This also explains why the review emphasizes narrative synthesis and certainty grading rather than pooled effect estimates.

Because only 13 heterogeneous studies were included and quantitative meta-analysis was not undertaken, publication and reporting biases were not formally assessed. Consequently, the possibility that unpublished or selectively reported studies may have influenced the available evidence cannot be excluded. Such issues become even more critical in the context of serious illnesses and death, where human intuition and empathy cannot be dispensed with.

Additionally, ethical and practical concerns surrounding AI-assisted communication training should be considered, including limitations in emotional realism, algorithmic bias, cultural sensitivity, and the potential risk of over-reliance on technology in emotionally complex palliative-care interactions.

### Implications for palliative‑care nursing education and practice

Despite these limitations, several practical implications for palliative-care nursing emerge. First, the review suggests that VR and AI should be considered adjunctive educational tools rather than replacements for established teaching methods. The use of scaffolded VR + SP models seems highly promising for palliative care situations. According to Hall et al. (2024), the combination of relatively inexpensive VR along with SPs and debriefing sessions will make it possible to significantly boost students’ confidence in communicating at the end of life stages, lowering the levels of anxiety without affecting emotional authenticity. For palliative care nurse teachers, this implies that they should leverage VR to mentally and emotionally prepare their students prior to role-playing and live patient encounters [9].

Second, VR-based EOL and post-mortem care modules can easily standardize instruction regarding the technical as well as relational skills required for EOL care, overcoming problems of variance in exposure and pre-existing proficiency [21, 22]. The use of such modules may prove especially useful in cultures where death is considered taboo and where patient contact is less readily available. This is certainly one of the clearest implications of the review, as it demonstrates the way in which immersion could assist in closing an educational gap where the availability of palliative exposure is not sufficient.

Third, interdisciplinary VR simulation can offer cost-effective methods for nurses to simulate interaction with physicians and other care providers when dealing with patients who are critically ill or dying, which is essential for palliative care practices [11, 17]. Used effectively, multi-user VR has the potential to act as a substitute for expensive live simulation without losing any of its value. Overall, the conclusion from the results is that the immediate benefit of education would be in improving basic communication skills, while specific adaptation to the field of palliative care remains a priority.

Fourth, AI-based virtual counseling and virtual-patient platforms provide flexible, low-barrier settings for practicing basic micro-skills of communication, which could be modified for palliative-specific applications in the future [13, 26, 27]. Careful co-development with palliative-care specialists, patients, and their family members will be key to making sure that these technologies demonstrate proper language use, empathy, and ethical awareness [6, 23].

Lastly, implementation studies emphasize that institutional support, faculty training, and proper curriculum design are crucial for the adoption of VR and AI, especially considering that the major obstacles include costs, technical challenges, and educators’ reluctance [6, 23]. Therefore, it is reasonable for palliative-care training programs to treat these innovations as complementary tools within a wider learning experience. In other words, the review provides useful guidance in terms of indicating areas of application of technology and where the evidence base is lacking.

### Recommendations for future research

Based on the findings of this systematic review, the following recommendations are proposed for future research:

**Conduct palliative‑specific RCTs and mixed‑methods evaluations** of VR and AI communication‑training interventions for practicing palliative‑care nurses and multidisciplinary teams, comparing these tools with established methods such as SP‑based workshops and bedside teaching [14, 17].

**Incorporate robust outcome measures**, including standardized patient ratings, validated communication checklists, and patient/family‑reported experience, alongside self‑efficacy and knowledge, to better capture educational impact [8, 13].

**Evaluate long‑term retention and transfer to practice**, tracking whether skills learned in VR and AI environments are sustained and used in real‑world palliative‑care encounters over time [5, 29].

**Design and rigorously test palliative‑specific AI conversational agents**, ensuring safety, ethical oversight, and transparent communication about limitations, and assessing their role as adjuncts to human‑facilitated training rather than replacements [6, 7, 31].

**Study the educational “active ingredients”** of AI/VR design—such as immersion level, debriefing structure, feedback type, and narrative design—to optimize future tools for palliative‑care communication training [6, 7, 31].

## Conclusion

Artificial intelligence- and virtual reality-based communication training represents an emerging and promising educational approach within nursing education. The available evidence demonstrates improvements in communication confidence, learner engagement, self-efficacy, communication performance, and educational satisfaction across a range of learning environments [9, 12, 17, 21, 22, 24, 25]. Although evidence directly related to palliative care communication remains limited, current findings support the integration of AI- and VR-based educational strategies as complementary approaches alongside traditional simulation and supervised clinical practice. Further well-designed randomized controlled trials with larger sample sizes and long-term follow-up are required to establish their effectiveness in palliative care nursing education and clinical practice [17, 25].

## Data Availability

No datasets were generated or analysed during the current study.
